# Unravelling factors associated with malaria parasitaemia among children 6–24 months to inform malaria interventions in Nigeria: evidence from 2021 Malaria Indicator Survey

**DOI:** 10.1186/s12936-023-04683-3

**Published:** 2023-08-28

**Authors:** Chinazo N. Ujuju, Olugbenga A. Mokuolu, Chinyere Nwafor-Okoli, Kenechi O. Nnamani

**Affiliations:** 1 Research Department, Data for Decisions Nigeria Ltd, Abuja, Nigeria; 2 Centre for Malaria and Other Tropical Diseases Care, University of Ilorin Teaching Hospital, Ilorin, Nigeria; 3 Department of Paediatrics, College of Health Sciences, University of Ilorin, Ilorin, Nigeria; 4 One Health Consortium, University of Calgary, Calgary, AB Canada; 5 Department of Paediatrics, Nnamdi Azikiwe University Teaching Hospital Nnewi, Nnewi, Anambra State Nigeria

**Keywords:** Malaria, Nigeria, Children, Parasitaemia, Cross-sectional study

## Abstract

**Background:**

As an additional two million malaria cases were reported in 2021 compared to the previous year, concerted efforts toward achieving a steady decline in malaria cases are needed to achieve malaria elimination goals. This work aimed at determining the factors associated with malaria parasitaemia among children 6–24 months for better targeting of malaria interventions.

**Methods:**

A cross-sectional study analysed 2021 Nigeria Malaria Indicator Survey dataset. Data from 3058 children 6–24 months were analyzed. The outcome variable was children 6–24 months whose parasitaemia was determined using a rapid diagnostic test (RDT). Independent variables include child age in months, mothers’ age, mothers’ education, region, place of residence, household ownership and child use of insecticide-treated net (ITN), exposure to malaria messages and knowledge of ways to prevent malaria. Logistic regression analysis was conducted to examine possible factors associated with malaria parasitaemia in children 6–24 months.

**Results:**

Findings revealed that 28.7% of the 3058 children aged 6–24 months tested positive for malaria by RDT. About 63% of children 12–17 months (aOR = 1.63, 95% CI 1.31–2.03) and 91% of children 18 to 24 months (aOR = 1.91, 95% CI 1.51–2.42) were more likely to have a positive malaria test result. Positive malaria test result was also more likely in rural areas (aOR = 1.79, 95% CI 2.02–24.46), northeast (aOR = 1.54, 95% CI 1.02–2.31) and northwest (aOR = 1.63, 95% CI 1.10–2.40) region. In addition, about 39% of children who slept under ITN had a positive malaria test result (aOR = 1.39 95% CI 1.01–1.90). While children of mothers with secondary (aOR = 0.40, 95% CI 0.29–0.56) and higher (aOR = 0.26, 95% CI 0.16–0.43) levels of education and mothers who were aware of ways of avoiding malaria (aOR = 0.69, 95% CI 0.53–0.90) were less likely to have a malaria positive test result.

**Conclusion:**

As older children 12 to 24 months, children residing in the rural, northeast, and northwest region are more likely to have malaria, additional intervention should target them in an effort to end malaria.

## Background

Despite advancements made globally through the implementation of international and national malaria control programmes, malaria still remains a global public health challenge with 247 million cases and 619,000 malaria-related deaths reported in the year 2021 [1]. With an additional two million malaria cases reported in 2021 compared to the previous year, concerted efforts toward achieving a steady decline in malaria cases are needed. Four countries account for 52% of all global malaria deaths with Nigeria accounting for 31% of the deaths [1]. Without accelerated action, the progress expected towards achieving malaria elimination according to the Global Technical Strategy for malaria may be unattained in 2030.

Over the years, pregnant women and children under five are most vulnerable to malaria, with severity and mortality higher among these vulnerable groups [2–5]. Although malaria control strategies target the general population, but intensive efforts focus on the most vulnerable population. In Nigeria, malaria control interventions include the use of insecticide-treated nets (ITN), prevention of malaria in pregnant women (IPTp), seasonal malaria chemoprevention (SMC) in children 6–59 months using sulfadoxine–pyrimethamine, and amodiaquine (SPAQ), prompt diagnosis of malaria cases and treatment with artemisinin-based combination therapy (ACT) [5]. These interventions have been effective in reducing morbidity and mortality due to malaria [6–8]. As such resulted in 45% decline in malaria prevalence from 42% to 2010 to 23% in 2018 [9]. However, the 2021 Nigeria Malaria Indicator Survey (NMIS) reported a slight decrease (4%) in malaria prevalence to 22% [10].

Without relenting in efforts to tackle the scourge of malaria, investments, innovations, new developments, and initiatives are being put in place to reduce the burden of malaria. In the Nigeria National Malaria Strategic Plan (2021–2025), Intermittent preventive treatment in infant (IPTi) now perennial malaria chemoprevention (PMC) evaluation pilot is proposed for states where seasonal malaria chemoprevention is not implemented. The World Health Organization (WHO) has recommended PMC be extended to children up to 24 months due to increased malaria prevalence among younger children [11]. In addition, WHO recommended RTS,S/AS01 malaria vaccine for the prevention of *Plasmodium falciparum* malaria in children living in regions with moderate to high malaria transmission will also be deployed to children in a four dose schedule starting from 5 months of age [11–13]. Deploying PMC and malaria vaccine in addition with existing interventions for malaria prevention would complement efforts to save children in Nigeria. Re-investigating malaria risk factors prior to deployment of new interventions is vital to inform effective delivery of new innovations for malaria prevention in children and achieve the leave no one behind ambition [14].

Prevalence of malaria and associated factors have been explored in pregnant women and delivering mothers [15–17]. Similar study has also been conducted in children under five and children up to 14 years old including neonates [18–22], and the general population [23]. Some studies have also explored prevalence of malaria in young children less than 6 months old [24, 25] and the first year of life [26]. This paper aim to explore factors associated with malaria parasitaemia in children 6–24 months old. Understanding factors associated with malaria parasitaemia in children are essential for better targeting of malaria control efforts.

## Methods

This study analysed cross-sectional survey data from 2021 NMIS. Survey was based on a nationally representative sample drawn from all states and local government area of the country. Analysis of NMIS dataset was conducted after adjusting for survey design cluster and non-response using the individual weight contained in the dataset. Population analysed was children 6–24 months. The outcome variable was children 6–24 months whose malaria parasitaemia was determined using rapid diagnostic test (RDT) during the survey. The independent variables included in the analysis include child’s age in months, mothers’ age, mothers’ education, region, place of residence, mothers’ exposure to malaria messages and her knowledge of malaria prevention. Protective factor against the parasite by using an insecticide-treated net (ITN) was explored by including household ownership, and child use of ITN as independent variables. These variables were chosen as previous studies have established their association with malaria parasitaemia [18, 20, 23, 27–31].

Both bivariate and multivariate statistical data analyses were performed using the statistical package Stata/SE 14.0. While bivariate analysis explored the malaria parasitaemia in comparison with the independent variables. The chi-squared test of independence was used to determine any significant associations between malaria parasitaemia and the independent variables. Variables with p-value < 0.2 at bivariate level were included into the logistic regression model and the unadjusted and adjusted odds ratio were derived to examine possible factors associated with malaria parasitaemia in children. Significant associations were measured at 5% alpha level (p < 0.05).

### Ethical consideration

This study utilized a population based NMIS dataset obtained from The Demographic Health Survey (DHS) Program, which was available online. The survey was approved by National Health Research Ethical Committee of Nigeria (NHREC) and ICF institutional Review Board. Informed consent was obtained from the caregivers of the children to participate in the survey and for blood sample collected from the children. The DHS Program followed regulations for guarding respondents’ privacy while gathering data. DHS also deleted all personally identifiable information from the online-accessible dataset. This study didn’t need any further ethical approvals because the DHS Program already requested and gained approval before the survey. Authors were given permission by the DHS Program to utilize the dataset for this study.

## Results

Descriptive analysis presents the sociodemographic characteristics of children 6–24 months and the prevalence of malaria parasitaemia. While bivariate and multivariate analysis determined the factors associated with malaria parasitaemia in children 6–24 months.

### Sociodemographic characteristics

A total of 3058 children 6–24 months were included in the study. Table 1 shows the socio demographic characteristics of these children. About 974 (31.8%) were aged 6–11 months; 1031 (33.7%) were aged 12–17 months while 1053 (34.4%) were aged 18–24 months old. Findings revealed that a higher proportion (51.1%) of their mothers were aged 25–34 years. About 43.2% of mothers had no education, while one out of three mother (30.8%) had secondary level of education. A higher proportion of the children (71.6%) reside in rural areas. While 2048 (67.0%) children live in households that had insecticide-treated nets (ITNs) almost half of the children (47.7%) slept under ITN the night before the survey. About 43.4% of mothers of these children were exposed to malaria messages in the last 6 months while 79.4% were aware of ways of preventing malaria.

**Table 1:** Sociodemographic characteristics and factors associated with malaria parasitaemia in children 6 to 24 months in Nigeria (N = 3058)

Variables	n (%)	Malaria positive to RDT	
		Crude OR (95% CI)	Adjusted OR (95% CI)
Child age (months)			
6–11 months	974 (31.8)	1.00	1.00
12–17 months	1031 (33.7)	1.70 (1.38–2.08)	1.63 (1.31–2.03)
18–24 months	1053 (34.4)	1.66 (1.32–2.09)	1.91 (1.51–2.42)
Mothers age			
15–24 years	820 (26.8)	1.00	1.00
25–34 years	1562 (51.1)	0.73 (0.59–0.90)	0.91 (0.73–1.13)
35 years and above	677 (22.1)	0.76 (0.58-1.00)	0.86 (0.65–1.13)
Mothers educational level			
No education	1322 (43.2)	1.00	1.00
Primary	469 (15.3)	0.66 (0.48–0.88)	0.76 (0.55–1.04)
Secondary	942 (30.8)	0.30 (0.23–0.40)	0.40 (0.29–0.56)
Higher	326 (10.7)	0.15 (0.10–0.24)	0.26 (0.16–0.43)
Mother heard/seen malaria messages			
No	1732 (56.6)	1.00	1.00
Yes	1326 (43.4)	0.72 (0.58–0.87)	0.95 (0.76–1.18)
Mother know ways to avoid getting malaria			
No	630 (20.6)	1.00	1.00
Yes	2427 (79.4)	0.55 (0.44–0.70)	0.69 (0.53–0.90)
Sex of household head			
Male	2868 (93.8)		
Female	190 (6.2)		
Household own ITN			
No	1010 (33.0)	1.00	1.00
Yes	2048 (67.0)	1.20 (0.96–1.51)	0.78 (0.58–1.06)
Child slept under ITN			
No	1601 (52.3)	1.00	1.00
Yes	1457 (47.7)	1.46 (1.16–1.83)	1.39 (1.01–1.90)
Region			
Northcentral	554 (18.1)	1.00	1.00
Northeast	506 (16.6)	1.85 (1.24–2.78)	1.54 (1.02–2.31)
Northwest	1082 (35.4)	1.93 (1.33–2.79)	1.63 (1.10–2.40)
Southeast	232 (7.6)	0.78 (0.46–1.32)	1.26 (0.69–2.30)
Southsouth	318 (10.4)	0.96 (0.60–1.52)	1.46 (0.85–2.49)
Southwest	365 (11.9)	0.46 (0.28–0.76)	0.96 (0.56–1.67)
Place of residence			
Rural	2191 (71.6)	1.00	
Urban	867 (28.4)	2.65 (1.97–3.56)	1.79 (1.36–2.36)

### Prevalence of malaria parasitaemia

**Fig. 1:**
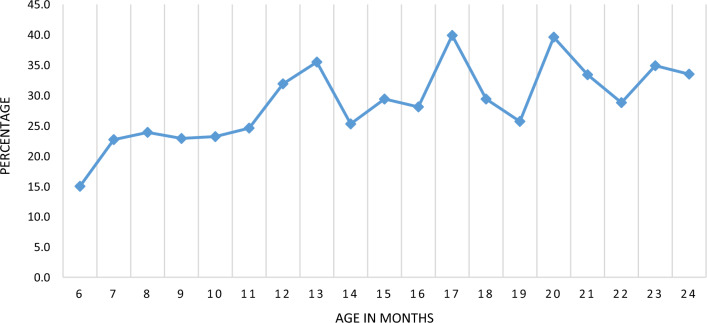
Prevalence of malaria parasitaemia in children 6–24 months using RDT

Data on 3058 children 6–24 months analysed revealed 878 (28.7%) of the children tested positive to malaria with RDT. Figure 1 shows prevalence of malaria by age in months. Results show an increase in the malaria cases from 6 months of age, which plateaus between 8 and 10 months and peaked at 13th months, 17th month and 20th months of age. Higher malaria cases were seen in the second year of life compared to the first year of life.

### Bivariate and multivariate analysis

Table 2 presents the bivariate analysis of the factors associated with malaria parasitaemia in children 6–24 months by sociodemographic characteristics. Findings show that malaria parasitaemia was associated with the age of child in months, age of mother, education of mother, region, place of residence, sleeping under ITN, mothers’ exposure to malaria messages in the past 6 months and knowledge of ways of preventing malaria (p < 0.05). While malaria prevalence in children 6–11 months was 21.9%. Malaria prevalence in children 12–17 months (32.1%) and 18–24 months (31.7%) were significantly higher (P < 0.000). Malaria parasitaemia was also significantly associated with place of residence (P < 0.000). About 33.7% of children in the rural area had a positive malaria test result compared to 16.1% in the urban area. Geographic variation was also observed with Northeast (36.3%) and Northwest (37.2%) having higher prevalence compared to other regions. Children of mothers with secondary (17.3%) and higher level of education (9.4%) were significantly less likely to have a positive test result compared to children whose mother had primary (31.2%) and no education (40.8%). Interestingly, about 32.8% (P < 0.001) of children whose mother reported that they slept under an ITN the night before the survey had a positive test result. While children of mothers’ who have seen or heard malaria messages (24.9%) and mothers’ who know ways of avoiding malaria (26.1%) were significantly less likely (p < 0.001) to have a positive malaria test result.

**Table 2:** Malaria parasitaemia in children 6 to 24 months by sociodemographic characteristics

Variables	Malaria parasitaemia in children 6–24 months		P value
	Negative n (%)	Positive n (%)	
Child age (months)			0.000
6–11 months	761 (78.1)	213 (21.9)	
12–17 months	700 (67.8)	332 (32.1)	
18–24 months	719 (71.3)	334 (31.7)	
Mothers age			0.01
15–24 years	545 (66.5)	274 (33.5)	
25–34 years	1144 (73.2)	418 (26.8)	
35 years and above	490 (72.5)	186 (27.5)	
Mothers educational level			0.000
No education	783 (59.2)	538 (40.8)	
Primary	322 (68.8)	146 (31.2)	
Secondary	779 (82.7)	163 (17.3)	
Higher	295 (90.6)	31 (9.4)	
Mother heard/seen malaria messages			0.001
No	1184 (68.3)	548 (31.7)	
Yes	995 (75.1)	330 (24.9)	
Mother know ways to avoid getting malaria			0.000
No	385 (61.1)	245 (38.9)	
Yes	1794 (73.9)	633 (26.1)	
Household own ITN			0.110
No	745 (73.8)	265 (26.2)	
Yes	1434 (70.0)	614 (30.0)	
Child slept under ITN			0.001
No	1200 (75.0)	401 (25.1)	
Yes	980 (67.2)	478 (32.8)	
Place of residence			0.000
Rural	1452 (66.3)	739 (33.7)	
Urban	727 (83.9)	140 (16.1)	
Region			0.000
Northcentral	424 (76.5)	130 (23.5)	
Northeast	323 (63.7)	184 (36.3)	
Northwest	680 (62.8)	402 (37.2)	
Southeast	188 (80.7)	45 (19.3)	
South south	246 (77.3)	72 (22.7)	
South west	320 (87.6)	45 (12.4)	
Total	2179 (71.3)	878 (28.7)	

Table 1 also presents result of the multivariable logistic regression analysis. Findings show that children 12–17 months were about 63% more likely to have a positive test result (aOR = 1.63, 95% CI 1.31–2.03) while children 18–24 months were 91% more likely to have a positive test result. (aOR = 1.91, 95% CI 1.51–2.42). Similarly, children whose mother have secondary (aOR = 0.40, 95% CI 0.29–0.56) and higher (aOR = 0.26, 95% CI 0.16–0.43) level of education were less likely to have a positive malaria test result. Children residing in rural area were about twice (aOR = 1.79, 95% CI 1.36–2.36) more likely to have a positive malaria test result compared to their counterparts in the urban areas. Similarly, children in the Northeast (aOR = 1.54, 95% CI 1.02–2.31) and Northwest (aOR = 1.63, 95% CI 1.10–2.40) regions were more likely to have a positive malaria test result compared to their counterparts in other regions. Children whose mother stated they slept under ITN the night before the survey were about 39% more likely to test positive to malaria (aOR = 1.39 95% CI 1.01–1.90) while children whose mother know ways to avoid malaria were less likely (aOR = 0.69, 95% CI 0.53–0.90) to have a positive malaria test result.

## Discussion

This study examined factors associated with malaria parasitaemia in children 6–24 months by conducting a secondary data analysis of 2021 NMIS dataset. The true prevalence of malaria in children has not been characterized and this is expedient with the epidemiological shift in population at risk to malaria [24, 32]. Findings highlight a malaria prevalence of 28.7% in children aged 6–24 months old using RDT, with prevalence higher in children more than 12 months compared to younger children. This finding confirmed increased malaria prevalence in children more than one year in Nigeria. Previous studies have also reported increased prevalence of malaria with increased age of persons in the population [30, 33, 34]. As prevalence of malaria in these children is higher than 10%, targeted malaria prevention approach is required for this age group. The National Malaria Strategic Plan articulates the countries malaria prevention and control strategies towards a malaria free Nigeria. The newly introduced tool RTS,S malaria vaccine is reported to result in significant reduction in clinical malaria, severe malaria and severe malaria anaemia [35]. Positioning this intervention across the country should be based on evidence on malaria prevalence and possible factors associated with malaria. This study which enables understanding of the factors’ association with malaria in children revealed child age, mother’s education, region, knowledge of ways to prevent malaria and place of residence were associated with malaria parasitaemia in children 6–24 months old. This finding is similar to several other studies. For instance, some studies have documented children residing in rural areas have higher prevalence of malaria than children in urban areas [21, 36]. This is attributed to dirty environments common in rural areas, which increase the likelihood of contacting malaria. Another study also showed that malaria is well acknowledged as a disease of poor communities common in rural areas [17, 37]. As progress made in reducing malaria prevalence in children is a measure to assess the achievement of leave no one behind global ambition [14], additional interventions such as malaria vaccine which has a high potential of reducing inequality of accessing existing intervention should target underserved children to protect them from malaria [38].

Several studies have confirmed sleeping under ITN protects from malaria with reduced prevalence in children who slept under ITN [18, 27, 29]. This study agrees with these findings as majority of the children who slept under ITN were protected, while about 39% of children who slept under ITN had a positive test result. The likelihood of children who used ITN to have malaria has been reported elsewhere [39]. Those that had a positive result could have been exposed to mosquitoes at a time when net was not used [27, 34, 40]. As maternally acquired protection from malaria will have waned after 6 months and a steady increase in malaria cases seen with increase in child age, danger of morbidity and mortality due to malaria need to be averted in these children. Social behaviour change communication (SBCC) has increased the impact of health intervention in hygiene, HIV and nutrition and can improve malaria prevention and treatment behaviour [41–43]. This study has also demonstrated the effect of behaviour change communication (BCC) on malaria as knowledge of malaria was associated with lower prevalence of malaria parasitaemia. As BCC has notably improved behaviour in several interventions, interpersonal communication at community and health facilities should target women with lower level of education whose children have a higher prevalence of malaria to increase their knowledge about malaria prevention and treatment. The identified factors associated to malaria prevalence presented in this study should be considered for better targeting of interventions.

### Strength and limitations

This study has a representative sample at regional and national level to guide malaria prevention strategy in children less than two years old and decision-making. Being a cross-sectional study, results captured point prevalence of malaria. As such seasonal trends in malaria transmission was not accounted for in this study.

## Conclusion

Older children 12 to 24 months and children residing in the rural areas, northeast and northwest region were more likely to have a positive malaria test result while children whose mothers have a higher level of education and know how to prevent malaria were less likely to have a positive malaria test result. Additional malaria intervention should target children under two years old at higher risk to malaria to attain malaria elimination goals.

## Data Availability

The datasets used for this work are available with permission in the Demographic Health Survey Program repository, at http://www.dhsprogram.com/data/dataset.

## Abbreviations

ACT: Artemisinin-based combination therapy
BCC: Behaviour change communication
DHS: Demographic Health Survey
IPTi: Intermittent preventive treatment in infant
ITN: Insecticide-treated net
NHREC: National Health Research Ethical Committee of Nigeria
NMIS: Nigeria Malaria Indicator Survey
PMC: Perennial malaria chemoprevention
RDT: Rapid diagnostic test
SMC: Seasonal malaria chemoprevention
SPAQ: Sulfadoxine–pyrimethamine and amodiaquine
WHO: World Health Organization
